# Comparative Study of the Method of Decorticating on Chemical Composition and Physicochemical Properties of Xyloglucan Extracted from *Tamarindus indica* L. Seeds at a Semi-Industrial Scale

**DOI:** 10.3390/polym17040498

**Published:** 2025-02-14

**Authors:** Valeria Espíndola-Sotres, Elsa Gutiérrez-Cortez, Abraham Mendez-Albores, Arturo Aguirre-Gómez, Alfredo Maciel-Cerda, Moustapha Bah, Araceli Ulloa Saavedra, Francisco Luna-Vázquez, María Eugenia Ramirez-Ortíz, Alejandra Rojas-Molina, Isela Rojas-Molina

**Affiliations:** 1 Doctorado en Ciencias Químico-Biológicas, Facultad de Química, Universidad Autónoma de Querétaro, Cerro de las Campanas S/N, Las Campanas, Centro Universitario, Santiago de Querétaro C.P. 76010, Querétaro, Mexico; valee.so10@gmail.com; 2 Laboratorio de Investigación Química y Farmacológica de Productos Naturales, Facultad de Química, Universidad Autónoma de Querétaro, Cerro de las Campanas S/N, Centro Universitario, Santiago de Querétaro C.P. 76010, Querétaro, Mexico; moubah@uaq.mx (M.B.); fjlunavz@yahoo.com.mx (F.L.-V.); rojasa@uaq.mx (A.R.-M.); 3 Unidad de Investigación Multidisciplinaria (UIM), Laboratorio de Procesos en Ingeniería Agroalimentaria, FES-Cuautitlán, Universidad Nacional Autónoma de México, Cuautitlán Izcalli C.P. 54714, Estado de México, Mexico; 4 Unidad de Investigación Multidisciplinaria (UIM), Laboratorio 14-A1, Ciencia y Tecnología de Materiales, FES-Cuautitlán, Universidad Nacional Autónoma de México, Cuautitlán Izcalli C.P. 54714, Estado de México, Mexico; albores@unam.mx; 5 Unidad de Investigación Multidisciplinaria (UIM), Laboratorio de Análisis de Investigación en Química Agrícola y Ambiental, FES-Cuautitlán, Universidad Nacional Autónoma de México, Cuautitlán Izcalli C.P. 54714, Estado de México, Mexico; aag@unam.mx; 6 Instituto de Investigaciones en Materiales, Universidad Nacional Autónoma de México, Ciudad de México C.P. 04510, Mexico; macielal@unam.mx; 7 Unidad de Investigación Multidisciplinaria (UIM), Laboratorio 16, Procesos de Transformación y Tecnologías Emergentes en Alimentos, FES-Cuautitlán, Universidad Nacional Autónoma de México, Cuautitlán Izcalli C.P. 54714, Estado de México, Mexico; araceli_ulloa@yahoo.com.mx; 8 Laboratorio Experimental Multidisciplinario (LEM 1), FES-Cuautitlán, Universidad Nacional Autónoma de México, Cuautitlán Izcalli C.P. 54740, Estado de México, Mexico; eugenia.ramirez@cuautitlan.unam.mx

**Keywords:** *Tamarindus indica* L., tamarind seeds, xyloglucan, mechanical separation, separation efficiency, physicochemical properties

## Abstract

Xyloglucan from *Tamarindus indica* seeds (TISs) is a polysaccharide widely used in the food, biomedical, and pharmaceutical sectors. Nevertheless, the challenge in future research for the food processing industry is to provide adequate knowledge regarding natural product extraction, chemical modifications, interactions, and potential applications according to sustainability issues. The goal of this work was to implement a sustainable method for xyloglucan extraction from TISs at a semi-industrial scale and carry out the characterization of this hydrocolloid, to compare the effect of the technique of decorticating of seeds on the chemical composition and physicochemical properties of xyloglucan. The TISs were decorticated using soaking (DS) and roasting (DR) methods, and, then, the xyloglucan was extracted applying a semi-industrial mechanical separation process. Subsequently, the extraction yield, chemical content, Fourier transform infrared analysis, color, morphology, molecular weight (MW), viscosity, texture, Z potential, particle size, and thermal properties were evaluated. Xyloglucan extraction from TISs at a semi-industrial scale was demonstrated for the first time. The xyloglucan yield by DR (44.04%) was significantly higher (*p* < 0.05) compared with DS (41.42%), while separation efficiency was similar in both methods (~97%). Significant differences (*p* < 0.05) in fat, ashes, crude fiber, calcium, total phenolic content, and antioxidant capacity in xyloglucan samples were observed by applying DS and DR. The method of decorticating promoted changes in the MW and particle size of xyloglucan samples, which were reflected in the viscosity, particle size, texture attributes, Z potential, and thermal properties of xyloglucan. These results show that the decorticating method is an important issue to be considered in the resultant chemical and physicochemical properties of xyloglucan extracted from tamarind seeds, for suitable applications of the xyloglucan in the food processing and pharmaceutical industries.

## 1. Introduction

Globally, it has been estimated that around 1.3 billion metric tons of annually generated food is wasted, representing approximately one-third of the total food produced [1,2]. For this reason, the generation of food waste and its transformation into value-added products (bioactive compounds, biobased plastics, and biofuels) constitute a global concern to develop sustainable processes for food production [3].

In 2020, the production of tamarind (*Tamarindus indica* L.) in Mexico was 55,752 metric tons [4], mainly destined for pulp extraction. This means that, in this agro-industrial activity, many by-products (shells and seeds) are generated without valorization [5].

The *T. indica* L. seeds represent ~30% of the total composition of this fruit [6], and they are used to obtain xyloglucan (galactoxyloglucan), which is a polysaccharide whose structure is made up of a β-(1,4)-D-glucan backbone substituted with side chains of α-(1,4) D-xylopyranose and (1,6) linked [β-D-galactopyranosyl-(1,2)-α-D-xylopyranosyl] to glucose residues [7].

The physicochemical properties of xyloglucan allow this hydrocolloid to be used in various technological applications such as stabilizer, thickener, viscosity enhancer, emulsifier, gelling, drug carrier, release retardant, and binder in the food and pharmaceutical industries [8].

Several studies have been reported for the xyloglucan extraction, where a sample pretreatment is carried out to eliminate the seed coat [9] and undesirable components (fats and proteins), which contaminate the final product [10,11,12]. Nevertheless, some of these procedures are very long and require no conventional heat treatments or the use of solvents that are not environmentally friendly. In addition, the yield of xyloglucan obtained is highly variable or not reported [13,14,15,16,17]. Consequently, all these factors have a significant impact on the processing cost [2]. On the other hand, the extraction processes have been carried out at the laboratory level (small scale). Thus, it is necessary to determine whether, on a larger scale, the operating conditions are adequate so that the production of xyloglucan can be profitable to obtain a value-added product [18].

Nowadays, innovations in recovering diverse molecules (compounds and natural products) from various sources are focused on the development of efficient, economical, and versatile techniques [19]. Regarding this, introducing magnetic ionic liquids (MILs) as an advanced pretreatment sample technique in microextractions allows high extraction efficiencies, good selectivities, and easy phase separations avoiding unnecessary steps such as centrifugation and filtration [20]. Aqueous two-phase systems composed of smart polymers (ATPSs) and thermo-separating magnetic ionic liquid aqueous two-phase systems (MILATPS) are other efficient, economical, and highly selective tools for the separation of target bioactive compounds and elimination of contaminants [21,22]. Despite the advantages that these emerging technologies offer, optimal operational parameters should be further explored to ensure the viability and functionality of isolated compounds.

Little attention has been given to the decortication of the *T. indica* seeds for the xyloglucan extraction. Therefore, investigation regarding the comparison of the decorticating method in yield and separation efficiency for xyloglucan extraction is still meager. In this context, it can be assumed that there is no universal protocol available for the xyloglucan extraction, which complies with all requirements for a sustainable process. This means that environmentally friendly solvents are used, easy solvent recovery methods are practiced, and minimal losses in the natural characteristics of xyloglucan are observed. For this reason, another aspect to consider is determining the possible applications of the products obtained from the sustainable extraction process, according to the characteristics that these products show [23,24].

We hypothesized that the method of decorticating *T. indica* seeds (soaking and roasting), before xyloglucan extraction, governs the properties of this biopolymer. Thus, the goal of this work was to implement a sustainable method for xyloglucan extraction from *T. indica* L. seeds at a semi-industrial scale and further characterize this hydrocolloid, to determine the effect of the procedure of decorticating seeds on the chemical composition and physicochemical properties of the extracted xyloglucan.

## 2. Materials and Methods

### 2.1. Xyloglucan Extraction

**Figure 1:**
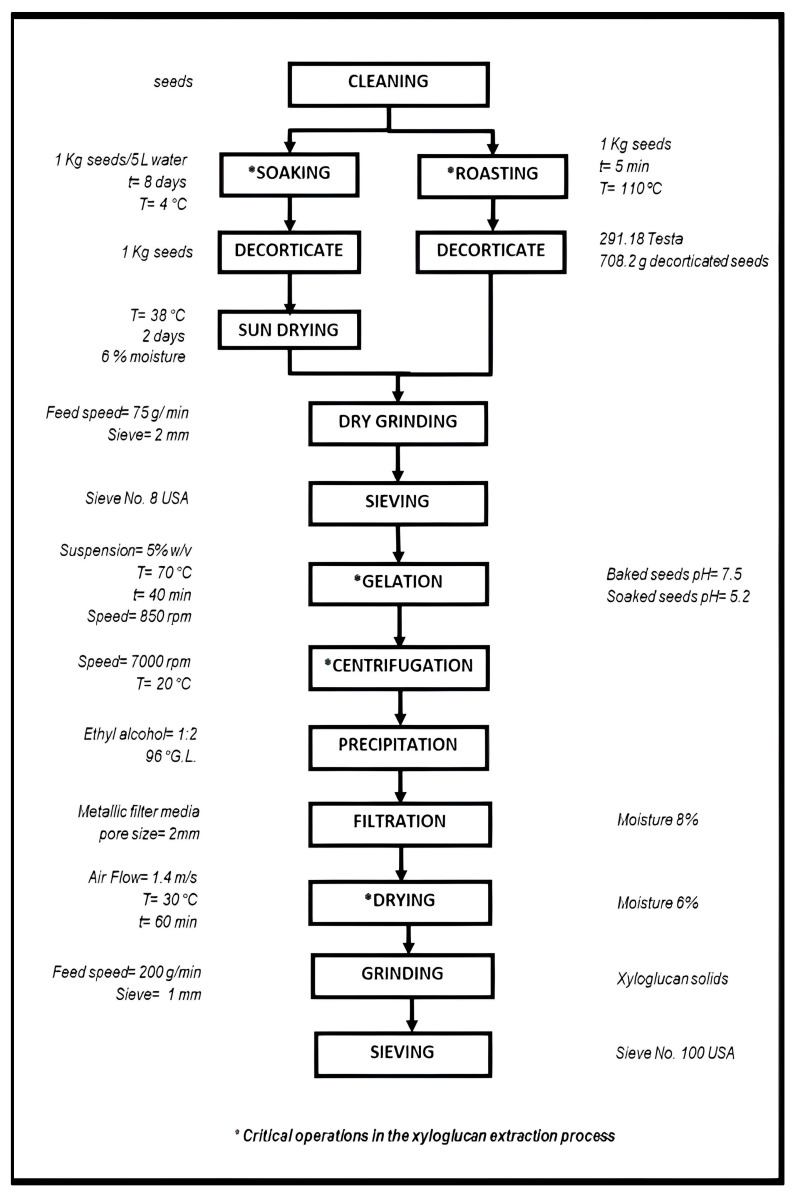
Block diagram for the xyloglucan extraction from *T. indica* seeds.

Xyloglucan extraction from *T. indica* seeds was carried out according to the block diagram shown in Figure 1. The decorticating method by the seeds soaking was carried out following the technique described by Khounvilay and Sittikijyothin [25] with minimal modifications. This method consisted of soaking 1 kg of seeds in 5 L of water at 4 °C for 8 days. Subsequently, the seed coat (testa) was removed, and the wet seeds were dried in a solar dryer at 38 °C for two days. Seed decortication by roasting was carried out by placing 1 kg of seeds on a perforated tray in an oven (Duke, model DUK-E102-G, Orldando, FL, USA) at 110 °C for 10 min and stirring the material every 5 min for uniform heating; then, the testa was removed from the seed.

The decorticated seeds were ground in a mill (Pulvex, mod. 200, Mexico City, Mexico) with a feeding rate of 75 g/min using a 2 mm sieve. Subsequently, the pulverized seed was passed through a sieve (No., 8 USA series, Retsch, Newtown, PA, USA). The material obtained was used to prepare 5% (*w*/*w*) suspensions. The suspensions were heated at 70 °C for 40 min, and the pH was determined. Next, the suspensions were centrifuged at 7000 rpm in a disk stack centrifuge (DIDACTA, Mod. TAG1/d, Torino, TO, Italy) to obtain soluble solids. Then, xyloglucan was precipitated from soluble solids by the addition of ethyl alcohol (96%) and water with a 1:2 (*v*/*v*) ratio. The separation of xyloglucan from liquid was carried out by filtration using a stainless-steel sieve with a pore opening of 2 mm. The xyloglucan retained in the sieve was dehydrated in an oven at 30 °C for 60 min. The material was pulverized with a blade mill (Pulvex, mod. 200, Mexico City, Mexico) at a speed of 200 g/min with a 1 mm outlet sieve. The solid granules of xyloglucan were passed through a sieve (No. 100 USA series, Retsch, Newtown, PA, USA) and stored until analysis. The ethyl alcohol used was recovered by distillation. The yield and separation efficiency were calculated using the equations reported by Quintero-García et al. [23].

### 2.2. Determination of Energy Consumption

The energy (E) required for xyloglucan grinding to obtain a powder was determined using the equation reported by Gutiérrez-Cortez et al. [26].

### 2.3. Compositional Analysis and Determination of Calcium Content

Dehydrated xyloglucan samples were analyzed following the methods established by the Association of Official Analytical Chemists (AOAC, 2000) [27]. The total carbohydrate content was calculated by difference (% Carbohydrates = 100 − [% moisture + % crude fiber + % crude protein + % ether extract + % ash]). In addition, the calcium content in xyloglucan samples was determined by the method (968.08) [27] with an atomic absorption spectrophotometer (Perkin Elmer, Mod. Analyst 300, Boston, MA, USA) and a flame detector.

### 2.4. Total Phenolic and Antioxidant Capability

The extraction of soluble phenolic compounds was carried out using 20 mL of a solution of water, methanol, and formic acid (18:80:02 *v*/*v*). The quantification of total phenolic content was conducted according to Li et al. [28] with the Folin–Ciocalteu reagent. The results were expressed as micrograms of gallic acid equivalents (µg GAE/g dry weight of sample). The antioxidant activity of xyloglucan was determined according to the method reported by Thivya et al. [29], and the results were expressed as Trolox equivalents (µg Trolox/g dry weight of sample).

### 2.5. Quantification of Galactose, Xylose, and Glucose by HPLC

The physicochemical properties of xyloglucans are highly dependent on their monosaccharide composition (i.e., galactose/xylose/glucose ratios); therefore, the quantification of galactose, xylose, and glucose was carried out. Initially, 1 g of each sample was hydrolyzed with 33 mL of HCl 4 M at a temperature of 100 °C for 8 h in a block heater (Thermo Scientific™, Waltham, MA, USA) [17,30]; then, the samples were centrifuged at 8000 rpm for 15 min, and the supernatant was filtered with a membrane and used to perform the determinations. The identification and quantification of monosaccharides was performed by a high-performance liquid chromatography (HPLC) system (Waters Co, Milford, CT, USA. Mod. e2695) coupled with a refractive index detector (Waters Co, Milford, CT, USA. Mod. 2414) and a Waters NH_2_ column (4.6 mm × 250 mm, 5 μm, Waters Co, Milford, CT, USA) at 80 °C. The mobile phase contained acetonitrile/water (80:20) in the isocratic mode and a total analysis time of 25 min. The sample injection volume was 20 μL, and the mobile phase flow rate was set at 1.5 mL/min. The retention times of samples were compared with monosaccharide standards (Sigma Chemical, Inc., St. Louis, MO, USA), and calibration curves were prepared for quantification. The analyses were conducted in triplicate.

### 2.6. Characterization by Fourier Transform Infrared (FT-IR) Spectroscopy

A Perkin Elmer Frontier NIR/MIR SP8000 spectrophotometer equipped with an ATR accessory (DuraSamplIR II, Smiths Detection, Warrington, UK) was employed for the analysis. The spectral range of 4000 to 400 cm^−1^ was chosen to cover the MIR region. To ensure robust outcomes, 32 scans were performed, resulting in improved spectrum quality and a favorable signal-to-noise ratio. A resolution of 4 cm^−1^ was selected to capture the relevant FTIR absorption bands. The bands were identified and analyzed using the Spectrum 10.4.2 software.

### 2.7. Color Measurement

The color values were recorded as L, darkness/lightness (0, black; 100, white); a (−a, greenness; +a, redness); and b (−b, blueness; +b, yellowness) using a colorimeter (Minolta, CR300, Tokyo, Japan). The color measurements were taken from five different positions [23].

### 2.8. Morphological Studies

Scanning electron microscopy (SEM) images of the xyloglucan samples were examined using a high-vacuum scanning electron microscope (JEOL, JSM-6060LV, Tokyo, Japan) with resolution in HV mode (5 nm). Each sample was carefully attached to a sample holder with carbon tape and sputtered with gold. The analysis conditions were 20 kV electron acceleration voltage and a 12–20 Pa pressure [23].

### 2.9. Estimation of Molecular Weight

The molecular weight (MW) was estimated by capillary viscometry following the methodology reported by Sixto-Berrocal et al. [31]. Briefly, 0.2 g of dry xyloglucan was dissolved in 100 mL of a buffer solution (0.3 M of HAc/0.2 M of NaAc) with pH ~4.6 and constant stirring for 24 h, and, then, the solution was filtered. Subsequently, xyloglucan dilutions were prepared using deionized water (0.025, 0.05, 0.1, and 0.2 gdL^−1^). The viscosity of each dilution was measured using an Ostwald-type capillary viscometer immersed in a water bath at 25 ± 1 °C. The fall times of the buffer solution (t_0_) and the biopolymeric solutions (t) were measured, and five measurements were performed for each sample. The relative, specific, and reduced viscosities were calculated to obtain the intrinsic viscosity (η). The average MW was estimated according to the Mark–Houwink Sakurada (MHS) equation, which relates the inherent viscosity of a polymer solution with the molecular weight of the polymer, using a value of *a* = 0.76 [32].

### 2.10. Viscosity Determination

The viscosity of samples was achieved in xyloglucan solutions prepared at concentrations of 1.5, 2.0, 2.5, 3.0, and 3.5% (*w*/*v*) at 25 °C with magnetic stirring until the hydration (~40 min) and the solutions were cooled at room temperature before the analysis. Measurements were performed at different shear rates in a range from 0 to 8 s^−1^ in a viscosimeter with a concentric cylinder geometry (Mettler-Toledo, Mod. RM180 Rheomat, Columbus, OH, USA) and a rotating cylinder sensor (DIN 1) [23].

### 2.11. Texture Analysis

Xyloglucan solutions were prepared at 5% (*w*/*v*) in distilled water at 35 °C using a thermo-shaker (Thermo Scientific, Mod. 4625Q, Waltham, MA, USA) at a rate of 250 rpm. The samples were stored until they reached a temperature of 25 °C. The force–time curves for two-cycle compression were obtained using a Universal Texturometer (Shimadzu, Mod. EZ-S, Kyoto, Japan). Then, the samples were placed in a Petri dish (diameter of 6.0 cm, height of 1.5 cm) and compressed with a cylinder (diameter 2.54 cm) at a rate of 1.3 mm/s to a compression depth of 8 mm (20% compression). When the compression stroke was completed, the plunger reversed and started its upward stroke. Subsequently, a second down and up-cycle was run on the same sample after 5 s. Five replicates were made for each sample.

### 2.12. Z Potential and Particle Size Measurements

Z potential (ζ-potential) and particle size measurements were performed with a particle size analyzer (ZetaSizer Pro, Malvern Instruments, Worcestershire, UK). Before the analysis, all samples were appropriately diluted using Milli-Q water (Millipore, Milli-Q Direct 8, Bay City, MI, USA) to yield an appropriate scattering intensity. Measurements were carried out at 25 °C using a DTS1070 disposable capillary cell (Malvern Panalytical, Westborough, MA, USA) and the particle size was obtained by calculating the average of five replicates. The Zeta potential was determined by electrophoretic light scattering. The conversion of the electrophoretic mobility to Zeta potential was performed using the Helmholtz–Smoluchowski equation [33]. Data were analyzed using the ZS Xplorer software v2.01 (Malvern Panalytical, Westborough, MA, USA).

### 2.13. Differential Scanning Calorimetry (DSC)

The thermal properties were analyzed utilizing a differential scanning calorimeter (Q100 TA Instruments, New Castle, DE, USA). Briefly, 5 mg of xyloglucan was placed into an aluminum standard pan (40 µL) and hermetically sealed. The plan was submitted to a heating ramp from −50 to 300 °C, with a heating rate of 10 °C/min, and nitrogen at a flow rate of 10 mL/min was employed. An empty pan served as a reference [34].

### 2.14. Thermogravimetric Analysis (TGA)

The mass loss of xyloglucan samples was evaluated through a TGA Q5000 (TA Instruments, New Castle, DE, USA) under the following conditions: a temperature range of 0–700 °C, a heating rate of 10 °C/min, and a nitrogen atmosphere with a flow of 10 mL/min. TA Universal Analysis software v4.5A was used for data processing [34].

### 2.15. Statistical Analysis

The results obtained from the xyloglucan extraction, chemical composition, and physicochemical characterization of experimental samples were analyzed by a *t* student’s test with α = 0.05, and, in each case, the statistical package SPSS version 2.2 was used.

## 3. Results and Discussion

### 3.1. Xyloglucan Extraction

Table 1 shows the yield, separation efficiency, and energy consumed by roasting and soaking *T. indica* seeds as decortication methods for xyloglucan extraction. The yield obtained in decorticated seeds with the roasting method (44.04 ± 0.37%) was significantly higher (*p* < 0.05) than the yield observed in the soaking method (41.42 ± 0.10%). Soaking is a culinary process to remove soluble components in legume seeds, i.e., antinutritional factors (α-galacto-oligosaccharides), which are eliminated in the discarded soaking solution [35]. In addition, soaking causes a decrease in carbohydrate contents (starch, total sugars, reducing sugars, total carbohydrates) as a result of the simple diffusion of sugars after being solubilized [36]; consequently, these losses have an impact on the yield. The yields of this current study are higher than those reported by Carvalho et al. [37], who obtained a yield of 7.7 and 32.66% decorticating seeds by soaking in water at 100 °C for 4 h using consecutive extractions with hot and cold water, respectively. Likewise, our yields are higher compared to those observed by Crispin et al. [11] and Alpizar-Reyes et al. [38], who reported values of 27.93 and 29.83%, respectively. All these authors carried out the xyloglucan extraction from tamarind seeds without coat removal. Regarding the yield obtained by the roasting method, Chawananorasest et al. [10] reported yields from 11.15 to 54.65% decorticating the seeds in an oven at 100 °C for 30 min, applying accelerated solvent extraction (ASE) and defatting tamarind seed powder using organic solvents (methanol and hexane) before the extraction of xyloglucan. The xyloglucan yield observed in our extraction process (44.04%) is in the range reported by these authors with the advantage that, in our case, the use of solvents is avoided, and the roasting time is also reduced according to the criteria for an eco-friendly process. Two decorticating methods involving 1) roasting seeds at 40 °C for 24 h and 2) the microwave heat pretreatment of seeds followed by soaking and hot air drying showed maximum yields of 22.12 and 25.8%, respectively [11,17]. Both procedures included defatting and protein soluble removal from the seeds to obtain a xyloglucan free of impurities. As more steps are involved in the xyloglucan extraction, the yield of xyloglucan decreases, as the processing cost increases. Thivya et al. [29] applied different extraction methods to obtain xyloglucan from roasted tamarind seeds, i.e., hot water extraction, cold water extraction, ultrasound-assisted extraction, microwave-assisted extraction, and alkali extraction, and, in all cases, the xyloglucan yield was less than 25%. In contrast, Limsangouan et al. [16] reported xyloglucan yields of 46.44, 53 and 62.28% from decorticated seeds, applying hot water extraction (conventional method before removal of fat and proteins), high-pressure assisted extraction, and super-critical water extraction, respectively. Definitively, higher yields are observed applying novel extraction methods; nevertheless, it is important to evaluate the feasibility of implementing a process easily adapted by small, medium, and large-scale producers, since greater yields for xyloglucan extraction imply increasing costs for manufacturers and issues from an ecological point of view [29].

No significant differences (*p* < 0.05) were detected in separation efficiency between soaking and roasting decorticate (see Table 1); therefore, the decorticating method does not influence this parameter. These values are similar to those reported by Quintero-García et al. [23], who also used a disk stack centrifuge, reporting a separation efficiency of 97% for *O. ficus indica* mucilage extraction. In this regard, the utilization of a disk stack centrifuge, commonly used in the food industry as a result of its mechanical simplicity, attains high G-force, allowing the mechanical separation between two phases with different densities efficiently [39].

The energy expenses to process 1 kg of tamarind seeds (from the first grinding, centrifugation, drying, and second grinding, including oven roasting for the roasting decortication method) were not significantly different (*p* < 0.05), using both decorticating methods since the unit operations for the seed processing after the pre-treatment step do not differ, as is shown in Figure 1. Nonetheless, it is important to mention that, in the pretreatment of seeds, the soaking decorticate method requires a large amount of water (5 m^3^/Tn of seed) and additional energy, since the seeds are soaked for 8 days at refrigeration temperature (4 °C). In contrast, with the roasting decorticate method, only the energy consumption for the raw material roasting would be considered (Figure 1). The data shown in Table 1 constitute an initial attempt to scale up the xyloglucan extraction using a semi-industrial disk stack centrifuge, maintaining geometric similarity between the equipment at a target scale. At present, most of the procedures reported for the xyloglucan extraction have been developed at scale [10,11,16,37,38,40]; this fact increases extra uncertainty during scale-up, avoiding identifying potential hazards and associated risks (important solvent requirements, high energy demands, improper control over process conditions). In addition, some still apply the pretreatment of samples using organic solvents and proteases. All of these issues are critical to improving production capacity and resource efficiency and reducing the environmental impact [41].

### 3.2. Chemical Analysis

The chemical composition of xyloglucan extracted by soaking and roasting is presented in Table 2.

No significant differences were observed (*p* < 0.05) in the protein content of xyloglucan extracted by both decorticate methods. However, these values are below the data reported by other authors [11,38,40], applying or not purification methods to tamarind seeds (defatting and alkaline protein extraction) before xyloglucan extraction. The minimum value reported for this component is 5.04% [16]. In contrast, the fat content in the xyloglucan extracted from decorticated seeds by soaking was significantly higher (*p* < 0.05), in comparison to the fat content in xyloglucan extracted from decorticated seeds by roasting. This difference can be attributed to the loss of fatty acids contained in the tamarind seed during roasting since it has been shown that these compounds are sensitive to heating [42]. In this regard, Ghafoor et al. [43] pointed out that concentrations of linolenic, linoleic, and oleic acids in unroasted *Salvia hispanica* L. seeds are higher than those from roasted ones, so they recommend that chia seeds should be heated at temperatures below or equal to 90 °C to preserve their fatty acid profile. Interestingly, the fat content reported by other authors [9,11,29,30,40] is higher than in Table 2. However, as expected, lower fat values are observed when applying seed defatting as a purifying method (0.10 and 0.38%) [11,16].

Ash content in xyloglucan obtained from soaked seeds decreased significantly (*p* < 0.05) compared to the ash content in the xyloglucan extracted from roasted seeds. This difference has been attributed to leaching solid matter in soaking water, which includes minerals [36]. These results are associated with the calcium content, which was significantly lower (*p* < 0.05) in xyloglucan obtained from soaked seeds than that detected in xyloglucan from roasted seeds. Luo et al. [44] also found an important reduction in certain minerals (Ca, Mg, K, and Na) in faba beans and azuki beans after soaking in distilled water for 6 and 12 h at room temperature.

The soaking of *T. indica* seeds promoted a significant increase (*p* < 0.05) in crude fiber content concerning the roasting of seeds (Table 2). This can be explained as follows: cereals and legume seeds contain phytates, which are associated with bivalent minerals (Ca, Mg, Fe, and Zn) forming a phytate–mineral complex [45]. During soaking, these plant products undergo an important reduction in phytate content at 30 °C for 24 h as a result of hydrolysis from endogenous phytase [46]; then, phytate degradation triggers the release of the minerals aforementioned from the phytate–mineral complex, migrating towards the cell wall, where the soluble pectins are linked to monovalent minerals [47]; nevertheless, the release of divalent minerals, specifically Ca, replaces the monovalent minerals, and the pectin becomes insoluble, forming a strong cell wall [48], therefore being part of the crude fiber, which is an approximation of the cell wall material content in the vegetables [49].

No significant differences (*p* < 0.05) were observed in the carbohydrate content of xyloglucan obtained from seeds decorticated by soaking and roasting (Table 2). These results are higher than those reported previously, with ranges of 62.3–89.56% [11,38,40] and consistent with the data reported by Limsagouan et al. [16]. These authors mentioned that xyloglucan is the main carbohydrate stored in the tamarind seeds; thus, low protein, fat, and ash contents in xyloglucan imply a high purity of the compound and, consequently, a high separation efficiency, as shown in Table 1.

Phenolic content and antioxidant capacity in xyloglucan obtained from seeds decorticated by soaking and roasting methods are shown in Table 2. Total phenolic compounds were higher (*p* < 0.05) in xyloglucan extracted from soaked seeds than roasted seeds. This can be attributed to degradation, oxidation, isomerization, decomposition, or polymerization reactions that are a result of the roasting process, which affects the phenolic content. Although moderate roasting conditions were used in this study, total phenolic content and antioxidant capacity were reduced. Nevertheless, lower temperatures and times during roasting were not enough to eliminate the seed coat.

Several studies have demonstrated that quercetin, rutin, catechin, or chlorogenic acid are thermolabile due to their hydroxylated structure [50]. In contrast, although the soaking treatment of the seeds was in aqueous media, this process was carried out at 4 °C and in a closed container, which decreases the probability of oxidation reactions or an increase in the solubility of phenolic compounds [51,52]. Interestingly, total phenolic compounds in xyloglucan obtained from soaked seeds are higher than those reported in xyloglucan obtained with various methods, involving hot water extraction [53], ultrasound-assisted extraction, microwave-assisted extraction, alkali extraction [9], extractions with subcritical water at different temperatures [16], and methods that include the defatted and deproteinization of the tamarind seeds [11]. Even, in some cases, phenolic compounds have not been detected in the xyloglucan extracted with the aforementioned methods [29,53]. About the antioxidant capacity, it was observed that this parameter was lower in xyloglucan extracted from roasted seed in comparison to xyloglucan obtained from soaked seeds, suggesting that phenolic compounds are mostly responsible for the antioxidant activity samples, which depend on media and heat treatment [16,54,55]. These results are in line with those of Sorourian et al. [56] and Maghsoudlou et al. [50], who pointed out that the antioxidant activity of polysaccharides depends on covalently linked phenolic compounds and other factors (i.e., polysaccharide solubility, sugar ring structure, molecular weight, the occurrence of positive or negatively charged groups and protein moieties).

Significant differences (*p* < 0.05) were detected in galactose, glucose, and xylose content between samples (Table 2); additionally, the galactose/xylose/glucose ratios were in the range reported by Nguyen et al. [17], for xyloglucan extracted form tamarind seeds using microwave treatment for seed decortication analyzed by HPLC. The differences in the glucose/xylose/galactose between experimental samples can be related with the decortication method, which modified physicochemical properties of xyloglucan as is explained in the subsequent sections.

### 3.3. Fourier Transform Infrared Spectroscopy (FTIR)

**Figure 2:**
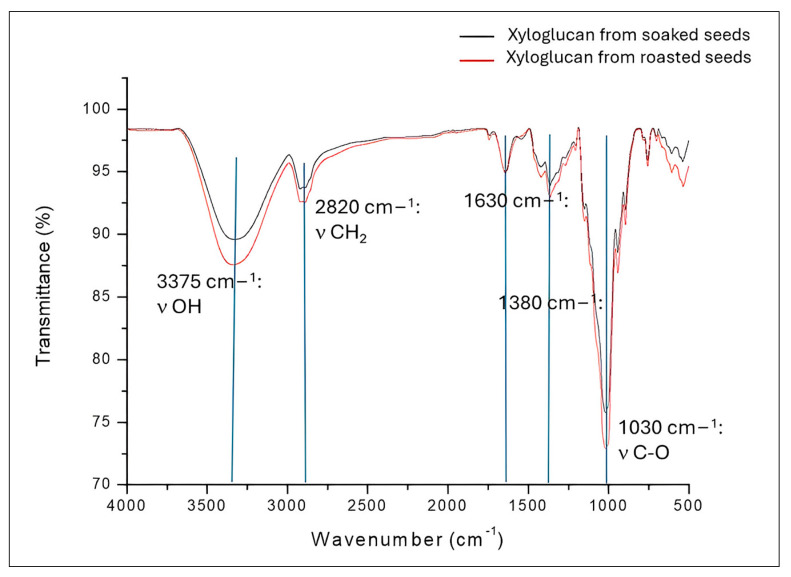
FTIR spectra of xyloglucan extracted from decorticated seeds by soaking and roasting.

The FTIR spectra of xyloglucan extracted from decorticated seeds by soaking and roasting are shown in Figure 2.

Similar bands were observed in the xyloglucan FTIR spectra of xyloglucan obtained by soaking and roasting, implying that the chemical structure of xyloglucan was not altered due to the decorticating methods (Figure 2). Significant bands in the current spectra are those due to the broad stretching band of OH (3375 cm^−1^), aliphatic C-H (2820 cm^−1^), and C-O (1030 cm^−1^), which have been reported previously for xyloglucan [10,11,30,36,42]. The peak at 1380 cm^−1^ has been considered characteristic of xyloglucan too [57], while the band at 1630 cm^−1^ can be attributed to the amide I (-C=O stretching) [58]. No stretching characteristic bands were identified for phenolic compounds; nonetheless, only a very weak band at 630 cm^−1^, ascribable to the C=C group of these phenolics was detected. Another spectral fact is that the most important bands increased their intensities from the xyloglucan of decorticated seeds by soaking to the xyloglucan of decorticated seeds by roasting. These increases can be attributed to a better xyloglucan yield obtained from roasted tamarind seeds because of xyloglucan losses from seeds during the soaking process (Table 1).

### 3.4. Color Analysis

Color is a quality attribute that determines the industrial application of raw materials. The color parameters of xyloglucan extracted from decorticated seeds by soaking and roasting are shown in Table 3.

The color parameters differed significantly (*p* < 0.05) between samples. The L* value in xyloglucan obtained from seeds decorticated by roasting decreased compared with that of xyloglucan extracted from seeds decorticated by soaking. In contrast, a* and b* values in xyloglucan from roasted seeds were higher than those observed in xyloglucan from soaked seeds. These results explain the darker color observed in xyloglucan obtained from roasted seeds. The color change in this sample can be explained by browning products derived from Maillard reactions, as a consequence of sugar interactions with amino acids from residual proteins during roasting [59]. These findings are in accordance with Limsangouan et al. [16], who detected an intense color in xyloglucan extracted using subcritical water. Color differences in xyloglucan determine its potential applications; for example, xyloglucan with a dark color can be used as an additive, in which the color of the final product is not a critical attribute for consumer acceptance. On the other hand, xyloglucan with a light color and that is translucent is ideal for biodegradable film development for food and pharmaceutical industries to obtain food packaging and pharmaceutical dosage forms.

### 3.5. Morphological Analysis

**Figure 3:**
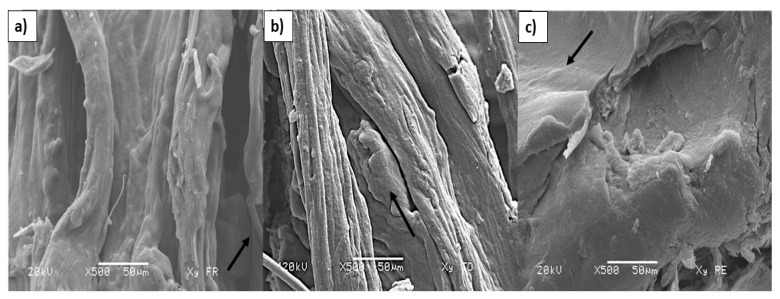
Micrographs of xyloglucan extracted from decorticated seeds by soaking (**a**,**b**) and roasting. (**c**) Detail of xyloglucan in the inner part of the fibrillar structures. Arrows in (**a**,**b**) denote the absence of fibers under the elongated structures and arrow in (**c**) denotes a smooth surface under the fibrillar structures.

Figure 3a,b show xyloglucan obtained from soaked and roasted seeds, respectively. In both cases, fibrillar and elongated structures are evident; though, in Figure 3a, these fibrillar structures are wider and less rough than in Figure 3b. In the inner part of the fibrillar structures (Figure 3a,b), these structures are not visible (see arrows), while a continuous matrix with a relatively smooth surface is detected as shown in Figure 3c (see arrow). Similar results were observed by Choi et al. [60] and Limsangouan et al. [30]. These authors showed long interconnected fibril structures, as well as a continuous structure slightly porous in xyloglucan extracted from tamarind seeds with boiling water (conventional method). On the other hand, Alpizar-Reyes et al. [38] observed spherical particles with a polymeric appearance in isolated xyloglucan from tamarind seeds and dried with a spay dryer. Differences in xyloglucan morphology are related to degradation processes of this polysaccharide, as a result of extraction and drying methods [19,30,38,60].

### 3.6. Estimation of Molecular Weight

The average molecular weight of xyloglucan obtained from tamarind seeds decorticated by soaking and roasting methods is shown in Table 3. The values are in the range of 115–2500 kDa reported by Nishinari et al. [61]. Nevertheless, the molecular weight of xyloglucan extracted from soaked and roasted seeds was higher than the maximum values reported previously (770.9, 770.89, and 699 kDa) [16,30,60]. The authors attribute low molecular weights to critical extraction conditions (subcritical water, high pressure, gamma ray, and electron beam irradiation), which caused major deterioration of the molecular chains in the xyloglucan components compared to xyloglucan extracted by conventional methods.

The molecular weight of xyloglucan from roasted seeds was significantly higher (*p* < 0.05) than that observed in xyloglucan from soaked seeds. The method of decorticating affected the molecular weight of xyloglucan, which agrees with Kazemi et al. [62]. These authors mentioned that the molecular weight of hydrocolloids depends on the extraction methods, solvents, and time. The low molecular weight in xyloglucan from soaked seeds can be explained by the action of β-glucanases, which are enzymes involved in the synthesis, remodeling, and turnover of cell wall components during physiological processes [63]. These enzymes cleave cellulosic and non-cellulosic polysaccharides containing contiguous (1,4)-β-glucosyl residues in their backbone, including xyloglucan [64]. The β-1,4-glucanases are involved in plant responses toward abiotic and biotic stresses as a defense mechanism [63]. Soaked seeds were exposed to abiotic stress, such as low temperature and excess moisture (see Figure 1), which probably triggered the activation of (1,4)-β-glucanases favoring xyloglucan depolymerization. Interestingly, the glucose, xylose and galactose content in xyloglucan from soaked seeds was significantly lower than those detected in xyloglucan from roasted seeds (Table 2). These data support that the soaking method promotes the loss of monosaccharides, decreasing the molecular weight of the xyloglucan from soaked seeds. Probably, a decrease in xylose and galactose contents also reduce the branched chains in the xyloglucan molecule extracted with the soaking decorticate method.

### 3.7. Viscosity Analysis

**Figure 4:**
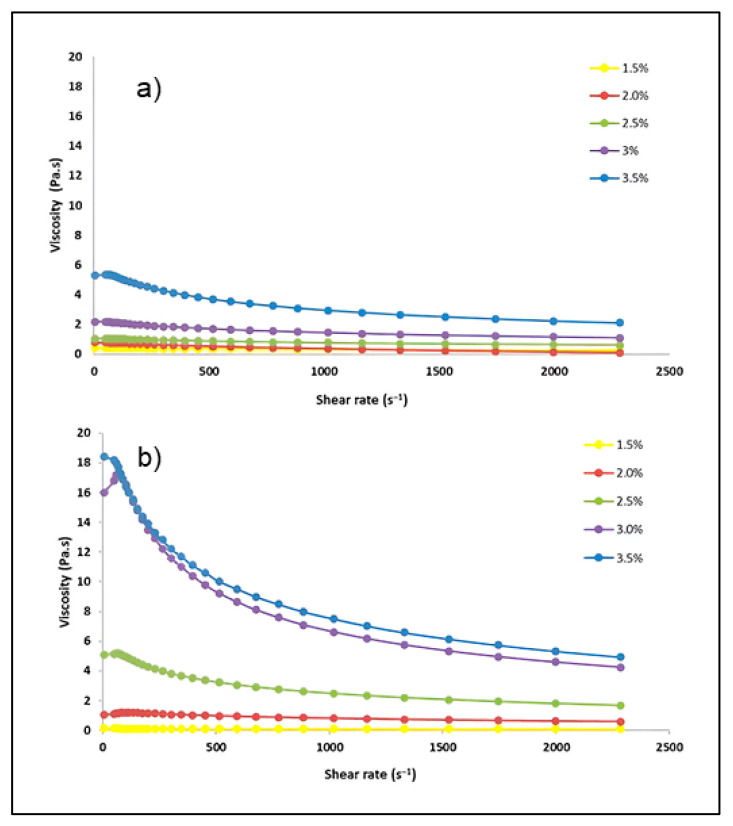
Viscosity values versus the shear rate of (**a**) xyloglucan extracted from seeds decorticated by soaking and (**b**) xyloglucan extracted from seeds decorticated by roasting at different concentrations (1.5, 2.0, 2.5, 3.0, and 3.5% *w*/*v*).

The viscosity values versus shear rates of xyloglucan extracted from seeds decorticated by soaking and roasting methods at different concentrations are shown in Figure 4a and Figure 4b, respectively.

In both cases, the xyloglucan at concentrations of 1.5 and 2% *w*/*w*, viscosity was independent of the shear rate obeying Newton’s law. On the contrary, at concentrations of 2.5, 3.0, and 3.5% *w*/*w*, the viscosity decreases as shear force increases, showing the behavior of a non-Newtonian fluid (pseudoplastic fluid). The effect of shear rate on viscosity is attributed to the progressive intermolecular network degradation; therefore, greater shear decreases the opposition of the molecules dispersed in the sample [65]. Likewise, the viscosity of dispersions increased as a function of the concentration and similar trends have been reported previously by other authors [11,30,37,66]. The viscosity of xyloglucan obtained from roasted seeds is higher than that observed in xyloglucan from soaked seeds, mainly at the highest concentrations (3 and 3.5% *w*/*w*). Frequently, the viscosity of hydrocolloid dispersions increases with molecular weight [67], which correlates with the molecular weight of xyloglucan obtained from roasted seeds, which is higher than its counterpart (Table 3). In branched polymers, the movement of the polymer molecules is prevented or at least drastically suppressed, since the branched chains reduce the translational diffusion coefficient of the polymer, and, consequently, the viscosity is increased, which is more shear rate-dependent than that of linear polymers [68]. Possibly, higher xylose and galactose contents (responsible of the branching pattern of xyloglucan) in xyloglucan from roasted seeds than in xyloglucan from soaked seeds explain higher viscosity values in xyloglucan extracted with the roasting decorticate method.

The viscosity values of xyloglucan at low concentrations (1.5 and 2% *w*/*w*) are comparable with the viscosity of xyloglucan extracted by the conventional method and applying spray drying in xyloglucan dispersions tested at the same concentrations [11,66]. However, the viscosity values observed in the present work are higher than the values reported for xyloglucan extracted by chemical, high-pressure, and subcritical water extraction methods [16,30,37]. Critical conditions applied during xyloglucan extraction with the aforementioned methods weaken the hydrogen bonds between molecules and leads to the xyloglucan chain reduction and, consequently, a decrease in viscosity [30].

Differences in the viscosity values of xyloglucan dispersions shown in Figure 4 determine their technological applications. For example, xyloglucan obtained by soaking seeds as a decorticate method can be used in pharmaceutical products with low viscosity (ophthalmological and nasal formulations) [8,69], as well as a thickening agent in juices and nectars [70]. On the other hand, xyloglucan from roasted seeds is adequate in film and coating development and as an excipient in gel formulation [71,72].

### 3.8. Texture Analysis

The texture attributes of xyloglucan gels obtained at 5% (*w*/*v*) are shown in Table 4.

In both cases, the xyloglucan samples developed soft gels, and, consequently, it was only possible to measure the parameters of hardness, springiness, and elasticity. Differences between xyloglucan samples in the aforementioned parameters were found to be significant (*p* < 0.05). It has been mentioned that xyloglucan forms a thermoreversible gel in an aqueous solution with the addition of small molecules and large amounts of saccharose and ethylic alcohol, due to these compounds together with xyloglucan developing a network structure in contrast with the native xyloglucan, which is presented as a viscoelastic solution [30,72,73]. Textural properties such as adhesion and hardness of composite gels are attributable to Van der Waals’ interactions, electrostatic forces, and molecular interactions via hydrogen bonds [74,75]. Most likely, the low values of textural parameters in xyloglucan from soaked seeds are a consequence of calcium losses (Table 2) and a low molecular weight as a result of possible xyloglucan depolymerization (Table 3), which reduce the molecular interactions between polysaccharide–polysaccharide chains and calcium–polysaccharide chains. It has been reported that the removal of galactose from tamarind xyloglucan promotes the gelation due to this molecular remotion increasing the ratio of hepta- and octa-saccharides and decreasing the ratio of nonasaccharides. The first saccharides do aggregate, and the last ones do not. This gelation is caused by the association of main chains by hydrophobic interaction; though, in aqueous solutions, xyloglucan molecules behave as almost flexible random-coil polymers and do not show any conformational change such as coil–helix transition, as seen in some other polysaccharides. This explains the soft gels developed by this polysaccharide [76].

Differences in the texture attributes of xyloglucans are useful in predicting their possible applications; for example, xyloglucan from roasted seeds is adequate for the development of food packaging and edible coatings, spreadable foods, and the formulation of mucoadhesive gels and films, while xyloglucan from soaked seeds is suitable for the manufacture of cosmetic and pharmaceutical products such as creams, suspensions, ophthalmic medications, and personal hygiene products.

### 3.9. Zeta Potential and Particle Size Determination

Zeta potential is a physical property, a magnitude of charge attraction or repulsion between particles, exhibited by any particle in suspension; i.e., it is the potential difference across phase boundaries between solids and liquids and is considered to be a key indicator of the stability of colloidal dispersions [77].

**Figure 5:**
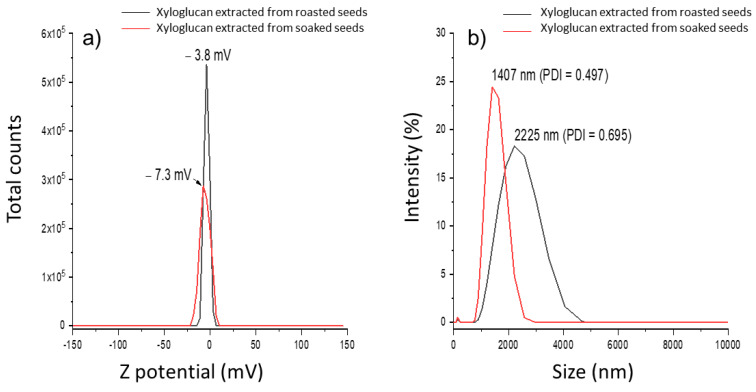
Representative graph of (**a**) the Zeta potential and (**b**) particle size distribution of the xyloglucan extracted from *T. indica* seeds. PDI = polydispersity index.

Statistical differences (*p* ≤ 0.05) were detected in the Z potential of the xyloglucan samples obtained by soaked and roasted seeds in their respective dispersions (Figure 5). However, the Z potential in the first case is in the range of −5 to −11 mV observed in xyloglucan extracted by conventional methods [11].

On the other hand, the Z potential in both samples is higher than the Z potential of mucilage extracted from O. ficus indica [19]; this can be explained as follows: (1) during extraction methods, the impurities, such as proteins that contribute to negative total charge, are removed (see Table 1) [11], and (2) the lack of carboxyl groups in the xyloglucan structure, which, in a pH range from 2.0 to 9.0, increase the negative charge of the Z potential for several biopolymers [78]. A relationship between viscosity and Zeta potential has been studied, and clear conclusions have not been reported; nevertheless, it has been established that there is an inverse relation between them. That is, the viscosity reaches its maximum value when the Zeta potential is zero (or trends to zero) and vice versa in mineral colloidal suspensions [79] and in colloidal apple juices added with hydrocolloids (carboxymethylcellulose and xanthan gum) [80]. Similar trends were observed in xyloglucan from roasted seeds, in which zeta potential is nearest to zero than xyloglucan from soaked seeds (Figure 5), and, in the first one, viscosities reached the highest values (Figure 4). These results suggest that xyloglucan extracted from soaked and roasted seeds can be used as a stabilizing, thickening, and gelling agent in the pharmaceutical and food industries. Furthermore, the negative Z potential values observed in both xyloglucan samples indicate possible interactions between this hydrocolloid and cationic biopolymers, i.e., proteins to obtain micro and nanoparticles by the complex coacervation method, with potential applications in controlled drug delivery systems [81].

The particle size distribution of the xyloglucan obtained from roasted seeds was significantly higher (*p* ≤ 0.05) than that observed for the xyloglucan from soaked seeds (2225 ± 42 and 1407 ± 151 nm, respectively); nonetheless, these values are above those reported previously (983.2 nm) [11]. High values of the polydispersity index (PDI > 0.5) imply less uniformity of the size distribution of particles [82]. Moreover, it has been pointed out that wide-molecular-weight distribution is due to a high proportion of long-chain xyloglucan molecules [30], and this agrees with molecular weights observed in the experimental samples (Table 3).

### 3.10. Thermal Analysis

The thermal properties of the xyloglucan samples were determined by thermogravimetric analysis (TGA) and differential scanning calorimetry (DSC). Results are shown in Table 5.

The TGA evidenced two stages of weight loss for both samples. The weight loss at the first stage has been associated with the loss of adsorbed and bound water [42,83], while the second one is related to xyloglucan decomposition [42,83,84]. Likewise, it has been pointed out that a weight loss between 100 and 200 °C relates to the loss of coordinated mineral compounds containing calcium [85]. Interestingly, the highest calcium content in experimental samples was observed in xyloglucan from roasted seeds (Table 2), which means that, during the soaking of tamarind seeds, a fraction of coordinated calcium compounds was removed, and, consequently, the weight loss was minor at this stage compared to xyloglucan from roasted seeds.

At the second stage of decomposition, the xyloglucan from soaked seeds showed a weight loss 10% greater than that observed for xyloglucan from roasted seeds (Table 5). In general, longer polymer chains tend to have greater intermolecular forces and crosslinking (hydrogen bonds) than shorter chains, which, in the first case, corresponds to a greater molecular order (greater crystallinity) [86]. This fact agrees with a higher molecular weight observed in xyloglucan from roasted seeds compared to that detected in xyloglucan from soaked seeds (Table 3). Xyloglucan from roasted seeds showed a lower mass loss up to 700 °C, residues of which were higher compared to those obtained for xyloglucan from soaked seeds (13.5 and 10.8%, respectively); this is according to the higher calcium content in the xyloglucan from roasted seeds (Table 2). Therefore, these data support that xyloglucan from roasted seeds is more resistant to thermal degradation than xyloglucan from soaked seeds. Probably, a higher glucose content in xyloglucan from roasted seeds than in xyloglucan from soaked seeds (Table 2) denotes the prevalence of long glucose linear chains, which show a higher thermal resistance than do short glucose chains in xyloglucan from soaked seeds.

The xyloglucan from roasted seeds shows a temperature of water evaporation starting at 50 °C and a maximum value at 112 °C, while the glass transition temperature was detected with a soft signal at 258 °C. On the other hand, xyloglucan from soaked seeds exhibits a temperature of water evaporation starting at 40 °C and a glass transition temperature at 254 °C with a maximum value of 97 °C. Interestingly, the glass transition temperature observed for experimental samples is similar to data reported by Marais et al. [87] and Del Real et al. [88] (252 and 242 °C, respectively). The enthalpy of vaporization of xyloglucan from roasted seeds was higher (236.9 J/g) than that detected for xyloglucan from soaked seeds (208.8 J/g). Likewise, the maximum temperature of water vaporization for xyloglucan from roasted seeds shows a difference of 11 °C to its counterpart. These results suggest that, in the xyloglucan from roasted seeds, there is a greater interaction between the polysaccharide linear chains, compared to the xyloglucan from soaked seeds, which provides greater rigidity to its structure.

The most important aspects to consider from the results derived from this study are as follows: (1) The yield observed for the xyloglucan extracted from roasted and soaked seeds was similar, (2) the total phenolic content and antioxidant capacity of xyloglucan from soaked seeds was higher compared with xyloglucan from roasted seeds, and (3) the MW, viscosity, Z potential, particle size distribution, textural properties (adhesiveness and springiness), and thermal resistance in xyloglucan from roasted seeds was higher than xyloglucan from soaked seeds. On the other hand, the xyloglucan yield observed in this current study, compared with those methods most recently reported in the scientific literature, was higher than that reported for xyloglucan extracted from tamarind seeds using microwave treatment for the seed’s decortication. The yield detected in our xyloglucan extraction method was similar to that observed by other extraction methods such as the high-pressure assisted process, ultrasound-assisted process combined with γ-irradiation, and electron beam irradiation. The super-critical water extraction method is one of the most efficient methods for the extraction of xyloglucan since the yields are greater than 50%, and the antioxidant capacity, as well as the total phenolic content, is the highest recently reported. The viscosity values and thermal stability of xyloglucan detected in the xyloglucan samples obtained with the two decorticating methods in the current study are higher than those reported with the aforementioned extraction processes. Our results are encouraging, since no methods reported at present have performed extractions on an industrial scale, and most of them do not mention the decorticating method used as a pre-treatment procedure of raw material.

## 4. Conclusions

In this current study, differences in the chemical composition and physicochemical properties of xyloglucan extracted from tamarind seeds decorticated by soaking and roasting methods were investigated. The yields and separation efficiencies obtained in this work were comparable with those observed previously by using different extraction methods, with the advantage that, in our case, the use of solvents and enzymes is avoided. In addition, the roasting time and, consequently, energy consumption are also reduced according to the criteria for an eco-friendly process. However, higher yields are indeed observed through innovative extraction technologies; in this work, the extraction of xyloglucan from tamarind seeds using a mechanical separation process on a semi-industrial scale was demonstrated for the first time. This fact is important to evaluate the feasibility of implementing a process easily adapted by small, medium, and large-scale producers since greater yields for xyloglucan extraction imply increasing costs for manufacturers and issues from an ecological point of view. The low contents of protein and fat in xyloglucan samples obtained from roasted and soaked seeds indicate that their purities are comparable to xyloglucan extracted by different extraction methods. The viscosity, molecular weight, textural properties (Hardness and springiness), Zeta potential, polydispersity index, and glass transition temperature of the xyloglucan extracted from soaked seeds were significantly lower than those of xyloglucan extracted from roasted seeds. These results support that the decortication method of *T. indica* seeds, as a pretreatment stage of raw material, influences the chemical composition and physicochemical properties of xyloglucan. This fact has not been studied in previous reports in this area and must be considered for suitable applications of xyloglucan in the food processing and pharmaceutical industries. Further studies are required, from an engineering point of view, for scale-up of the xyloglucan extraction at an industrial level, applying the soaking and roasting methods for the seeds decorticating.

## Figures and Tables

**Table 1:** Xyloglucan yield, separation efficiency of the xyloglucan obtained from *T. indica* seeds by using two decortication methods. Total energy consumption and energy cost of the processes.

Decorticate Method	Xyloglucan Yield (%)	Xyloglucan Separation Efficiency (%)	Total Energy Consumption * (kW/kg Dry Raw Material)	Energy Cost USD/kW·h
Soaking	41.42 ± 0.10 ^a^	97.36 ± 0.70 ^a^	15.82 ± 1.90 ^a^	2.27
Roasting	44.04 ± 0.37 ^b^	97.11 ± 0.50 ^a^	16.18 ± 0.30 ^a^	2.31

* Energy consumption includes the first grinding, centrifugation, drying, and second grinding, including oven roasting for the roasting decortication method. Values represent the mean ± standard deviation (SD), *n* = 5. Means in columns with different letters differ significantly (*p* < 0.05).

**Table 2:** Chemical proximate analysis, total phenolic content, antioxidant capacity, and monosaccharide content (galactose, xylose, and glucose) of xyloglucan obtained by using two decorticate methods (dry mass).

	Decorticate Method	Soaking	Roasting
Component			
Protein (%)		3.94 ± 0.10 ^a^	3.86 ± 0.15 ^a^
Fat (%)		0.69± 0.01 ^a^	0.26 ± 0.10 ^b^
Ashes (%)		0.47± 0.02 ^a^	0.70 ± 0.02 ^b^
Crude fiber (%)		1.26± 0.08 ^a^	1.17 ± 0.12 ^b^
Carbohydrates (%)		93.6± 0.90 ^a^	93.95 ± 0.70 ^a^
Calcium (mg/g)		1.58 ± 0.10 ^a^	1.72 ± 0.12 ^b^
Total phenolic content (µg GAE/g)		132.13 ± 11.95 ^a^	77.27 ± 9.79 ^b^
Antioxidant capacity (µg Trolox/g)		105.48 ± 8.72 ^a^	52.88 ± 5.24 ^b^
Galactose (%)		6.95 ± 0.14 ^a^	9.02 ± 0.10 ^b^
Xylose (%)		22.50 ± 0.87 ^a^	28.10 ± 1.05 ^b^
Glucose (%)		32.03 ± 1.2 ^a^	43.47 ± 0.65 ^b^
Galactose/xylose/glucose ratio by mass		1.0:3.2:4.6	1.0:3.1:4.8

The values represent mean ± standard deviation (SD), *n* = 5. Means in rows with different letters differ significantly (*p* ≤ 0.05).

**Table 3:** Color analysis and molecular weight of xyloglucan obtained by using two decorticate methods.

Decorticate Method	L*	a*	b*	Molecular Weight (kDa)
Soaking	+96.66 ± 0.10 ^a^	+0.20 ± 0.01 ^a^	+1.88 ± 0.15 ^a^	816.50 ± 0.35 ^a^
Roasting	+92.31 ± 0.12 ^b^	+2.28 ± 0.16 ^b^	+10.96 ± 0.12 ^b^	1926.97 ± 0.13 ^b^

The values represent mean ± standard deviation (SD), *n* = 5. Means in columns with different letters differ significantly (*p* ≤ 0.05).

**Table 4:** Instrumental texture attributes in xyloglucan obtained by soaked and roasted seeds of *Tamarindus indica*.

Sample	Hardness (N)	Springiness (%)	Adhesiveness (N)
Soaked seeds	1.39 ± 0.04 ^a^	51.96 ±1.84 ^a^	0.036 ± 0.007 ^a^
Roasted seeds	19.66 ± 1.78 ^b^	144.45 ± 3.87 ^b^	−0.852 ± 0.001 ^b^

Values represent the mean ± standard deviation (SD), *n* = 5. Means in columns with different letters differ significantly (*p* ≤ 0.05).

**Table 5:** Derivative thermogravimetric, residue content, and glass transition (Tg) of xyloglucan obtained by soaked and roasted seeds of *Tamarindus indica*.

Sample	Stage 1				Stage 2				
	T Range °C	Weight Loss %	T Peak °C	ΔH (J/g)	T Range °C	Weight Loss %	T Peak °C	Residues at 700 °C %	Tg °C
Soaked seeds	44–171	7.19 ± 0.36 ^a^	93.63	208.8	392–431	76.88 ± 2.1 ^a^	330.10	10.8 ± 0.20	254
Roasted seeds	42–138	9.16 ± 0.86 ^a^	112.21	236.9	302–398	66.32 ± 1.3 ^b^	326.96	13.5 ± 0.50	258

Means in columns with different letters differ significantly (*p* ≤ 0.05).

## Data Availability

The original contributions presented in this study are included in the article. Further inquiries can be directed to the corresponding authors.
